# Reaching accuracy declines with postural demand during whole-body leaning

**DOI:** 10.3389/fspor.2026.1843450

**Published:** 2026-06-09

**Authors:** Taisei Fujimura, Shota Hagio, Motoki Kouzaki

**Affiliations:** 1 Laboratory of Neurophysiology, Graduate School of Human and Environmental Studies, Kyoto University, Kyoto, Japan; 2 Laboratory of Motor Control and Learning, Graduate School of Human and Environmental Studies, Kyoto University, Kyoto, Japan

**Keywords:** balance control, fall avoidance, motor control pattern, postural threat, sensorimotor control, voluntary movement, whole body action

## Abstract

**Introduction:**

Whole-body movements in everyday tasks challenge the neuromuscular system. Prior studies have shown that appropriate postural control supports both task success and postural balance during quiet standing or under modest postural demands. However, it remains unclear how the central nervous system controls whole-body posture under high-demand conditions, such as maximal leaning postures. Here, we investigated how varying postural demands influence task performance in whole-body tasks.

**Methods:**

We used an upper-body reaching task in which participants leaned their upper body mediolaterally to reach a target without falling. Our task required various upper-body positions and accelerations by varying target positions and time constraints to reach a target. Therefore, our task induced participants to different magnitudes of destabilizing torque, which is defined as postural demand. Task performance in response to different target positions and time constraints was evaluated based on reaching accuracy.

**Results:**

Target position, time constraint, and movement distance significantly affected reaching accuracy. Specifically, participants could accurately reach targets requiring upright or moderately leaning postures. However, when targets required greater leaning postures, participants failed to reach them. Furthermore, the detrimental effects of shorter time constraints and longer movement distances on reaching accuracy became more pronounced when target positions required greater leaning postures.

**Discussion:**

These findings demonstrate that reaching accuracy was preserved under low to modest postural demands, but deteriorated once postural demands became high.

## 1 Introduction

Maintaining a standing posture is fundamental to daily life, as falls can lead to serious injury or worse. However, there are situations in which we intentionally accept the risk of falling. For example, in basketball, when a ball is about to go out of bounds, a player must catch it while keeping their feet inside the court. In such scenarios, the whole-body posture must deviate from the upright position to reach the ball; however, excessive leaning can result in a fall (1). Thus, large leaning postures are associated with an increased risk of falling. Although the central nervous system (CNS) is thought to account for the risk of falling when generating whole-body movements (2), the mechanism by which the CNS estimates and accepts fall risk remains poorly understood.

Postural balance relies on effective CNS control (3, 4) in the presence of several destabilizing factors. For instance, sensorimotor noise (5, 6) and internal torque from arm movements (7, 8) can threaten balance, even during quiet standing or simple arm raising in an upright posture. Additional task demands further complicate balance control, as greater trunk movement increases gravitational torque and reduced stability limits constrain whole-body movements (9, 10). Previous studies have demonstrated that the CNS can adapt remarkably well to such challenging conditions (11–14). For example, endpoint accuracy in arm-reaching during standing remains comparable to that observed in less challenging conditions, such as seated arm-reaching (15). These studies have generally focused on how the CNS compensates for destabilizing factors. Thus, the examined postural orientations were limited to upright or moderately leaning postures (16). Therefore, when higher postural demands, such as maximum leaning posture, are required, the control strategies adopted by the CNS remain unclear. If the CNS considers the risk of falling when generating movements (2), it may prioritize fall avoidance over achieving the required maximum leaning posture accurately. To understand the postural control mechanism comprehensively, it is important to characterize task performance in response to various postural demands, including maximum leaning posture.

With this motivation, we constructed a novel task in which participants reached a target using their upper body under varying postural demands. In this study, postural demand was defined by the magnitude of the destabilizing torque, that is, the net torque acting to deviate the body segments from the vertical axis. Participants were required to lean medio-laterally to control an upper-body control point, defined as the averaged motion from the head to the pelvis. The displacement and acceleration of the upper-body control point from the upright position modulate the destabilizing torque. Specifically, as the upper-body control point is far from the upright position, the destabilizing torque increases because gravity acts to rotate each body segment away from the vertical axis (9, 10). Moreover, greater leaning acceleration is associated with larger torques that drive the body away from the vertical axis, thereby increasing destabilizing torque (9). Thus, in order to manipulate the destabilizing torque, we systematically varied target positions and time constraints to reach a target, leading to varying displacement and acceleration of the upper-body control point. Targets located farther from the upright position required larger postural displacement. Additionally, acceleration was indirectly modulated by shorter time constraints, which necessitated higher velocities. This was because requiring an arbitrary acceleration directly was difficult. Together, by varying target position and time constraints, we systematically manipulated the destabilizing torque. Our study employed an upper-body reaching task rather than tasks based on center-of-mass or center-of-pressure displacement (17). This design encouraged participants to lean their body, rather than rely on strategies without greater destabilizing torque (e.g., only lower-body movements or shear forces control to the force plate).

The interest of our study is task performance under conditions requiring maximal leaning posture. However, it is difficult to predetermine each participant's maximum leaning posture, as it is unclear what determines the posture (e.g., muscle strength, body size, or neurophysiological factors) (18, 19). To address this issue, we designed the task to cover a wide range of postures, from easily achievable postures (e.g., upright standing) to those that could not be achieved without falling. This ensured that each participant's maximum leaning posture was included in the experiment.

This study aimed to characterize task performance under varying postural demand conditions, including maximal leaning postures. We investigated how postural demands affect task performance by comparing reaching accuracy in response to different target positions and time constraints. Characterizing task performance is important for understanding the control mechanisms during goal-directed whole-body movements.

## 2 Materials and methods

### 2.1 Participants

Twenty healthy adults [all males, age: 23.2 ± 1.4 years, height: 1.71 ± 0.01 m, body mass: 62.0 ± 5.9 kg, mean ± standard deviation (SD)] participated in this study. Participants approximately 1.70 m in height were recruited to control the effect of the body's physical properties. Consequently, the mean height of participants was 1.71 m (range: 1.68–1.73). All participants had normal or corrected-to-normal vision and no history of musculoskeletal or neurological disorders. The participants provided written informed consent prior to the experiment. This study was conducted by the Local Ethics Committee of the Graduate School of Human and Environmental Studies, Kyoto University (Approval Number 21-H-14).

### 2.2 Experimental setup

**Figure 1:**
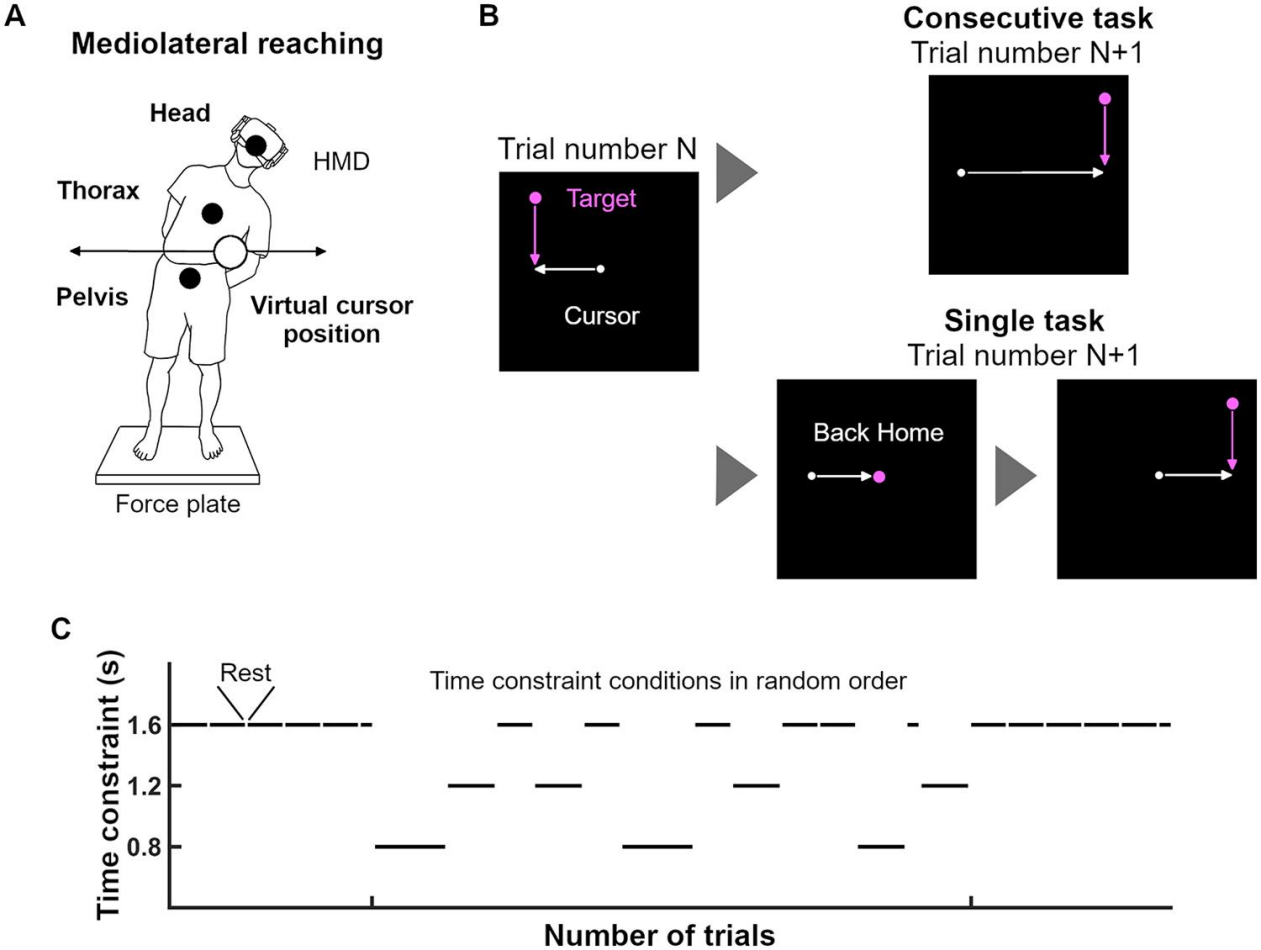
Experimental setup. **(A)** Participants stood on a force plate and swayed mediolaterally while wearing a head-mounted display (HMD). The motion capture system acquired the mediolateral positions of the participant's head, thorax, and pelvis to transform the mean position to the cursor position. **(B)** The screen was displayed through the HMD in front of the participants in virtual reality. Targets appeared at a fixed vertical height and moved downward. Participants swayed mediolaterally to move the cursor along a fixed horizontal path to reach the target. The mediolateral positions of the targets were at random and evenly distributed from side to side of the screen. In the consecutive task, the target appeared immediately after the previous one. In the single task, the target appeared at the center of the screen (home position), and the next target appeared once the cursor was within 1 cm of the home target. **(C)** Participants completed 2,000 trials, including two single tasks and three consecutive tasks. After completing the single task for 400 trials (pre-single phase), participants performed the three consecutive tasks for a total of 1,200 trials (consecutive phase) and then completed the single task for 400 trials again (post-single phase). Each single task included only the 1.6-second time constraint condition. The consecutive task consists of three time constraint conditions of 0.8, 1.2, and 1.6 s. Each condition had a total of 400 trials, divided into multiple blocks. The blocks in the consecutive task were presented in random order for each participant.

Participants stood barefoot on a force plate (TF-4060, Tec Gihan Co. Ltd., Kyoto, Japan) with their feet positioned parallel and 30 cm apart, defined by the distance between the lateral edges of the feet. The foot position was marked to ensure a consistent stance throughout the experiment. The position of the center of foot pressure (CoP) was calculated based on the force plate data at 1,000 Hz. Participants wore a head-mounted display (HMD; VIVE Pro 99HANW009-00, HTC, Taoyuan, Taiwan) to interact with a virtual reality (VR) environment (Figure 1A). The VR environment was used to eliminate task-irrelevant visual information. A screen (175 cm × 118 cm, 60 Hz refresh rate) was placed 2.3 m in front of participants in the VR environment.

Six body landmarks were tracked using a 3D optical motion capture system at 100 Hz (OptiTrack V100, Natural Point Inc., Oregon, USA): (1) right earmuff of the HMD, (2) left earmuff of the HMD, (3) the xiphoid process, (4) the vertebral prominence of the seventh thoracic vertebra, (5) the midpoint between both anterior superior iliac spines, and (6) the sacrum. The motion data were streamed in real time to LabVIEW (National Instruments, Austin, TX), where the six mediolateral positions were averaged, converted into cursor positions, and displayed on the screen. Consequently, the cursor on the screen (diameter: 3.2 cm) moved only in the mediolateral direction, corresponding to the mean position across the head, thorax, and pelvis (i.e., upper-body control point). Note that the six body landmarks were located symmetrically on opposite sides of the bod*y* axis, so that averaging these positions could cancel the body-twisting effects in the yaw axis.

More detailed technical setups are provided in the Supplementary Materials.

### 2.3 Whole body task

Participants performed a whole-body task to investigate how postural demands affect task performance. Postural demand was defined by the magnitude of the destabilizing torque, which was modulated by two factors: (1) the degree of deviation of the upper-body control point from the upright posture (9, 10, 18), (2) the acceleration of deviation (9, 10). The task manipulated the two factors by varying target positions and the time constraints to reach a target.

Before the task, participants were asked to stand upright on the force plate and the cursor position when participants stood upright was set at the center of the screen, referred to as the home position. Additionally, the line on which the cursor moved mediolaterally is referred to as the cursor movement line. In each trial during the task, a target (diameter: 9.6 cm) appeared at 22.4 cm above the cursor movement line and moved downward at constant velocity. The target disappeared when the target arrived at the cursor movement line. Participants were asked to control the cursor to reach the target while keeping their feet flat (Figure 1B left). In our task, the cursor corresponded to upper-body movement rather than the center of mass or CoP movement to prevent cursor control without large destabilizing torque (e.g., cursor control via only lower-body movements or shear forces on the force plate) (17).

To manipulate postural demand during the task, we varied the mediolateral target positions. For example, reaching a target far from the home position required participants to deviate their upper body from the upright posture. Therefore, the magnitude of the destabilizing torque increases due to the gravity (9, 10). To manipulate postural demand, we also varied the time constraints to reach a target. The time constraint was defined as the time interval between the target's appearance and disappearance. Therefore, the shorter time constraint required participants to lean with the greater velocity to cover the same distance, leading to greater acceleration. Since the target always moved the same distance, the time constraint was manipulated by changing its constant downward velocity. Thus, participants could visually perceive the remaining time in each trial from the target's downward movement.

**Figure 2:**
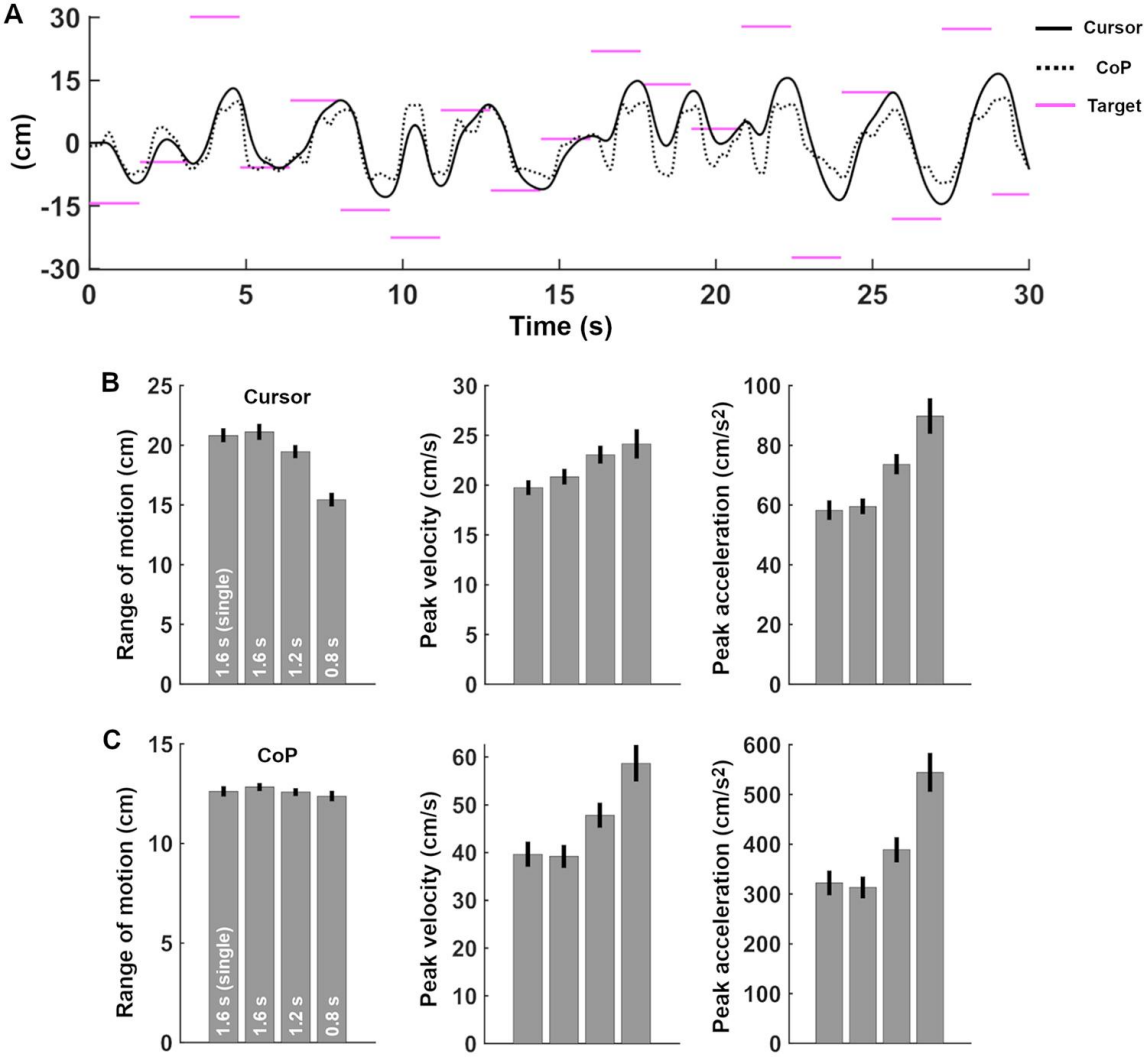
Trajectories and movement kinematic parameters. **(A)** Representative time series of cursor and CoP trajectories for the 1.6-second condition in the consecutive task. The solid black line indicates cursor movement; the dashed line indicates CoP movement. Each pink horizontal line represents a target position. **(B)** Group means of cursor range of motion, peak velocity, and peak acceleration across all conditions. Values are mean ± SEM across participants. Range of motion values were presented as absolute values, collapsed across left and right directions. **(C)** Group means of CoP range of motion, peak velocity, and peak acceleration across all conditions. Values are mean ± SEM across participants.

Given that the home position was zero, the mediolateral target positions ranged from ±30.8 cm to the left and right. This range was determined through a pilot study to include target positions unreachable without lifting a foot to induce near-falling situations. To cover the entire range, the range was divided into 40 target areas, and 10 target positions were randomly selected within each area. Consequently, a total of 400 targets were used in each task condition (see below). Target positions were presented in a random order within ±30.8 cm (Figure 2A).

In the experiment, there were two task types: single task and consecutive task. In the single task, after each trial, the target returned to the home position before the next one appeared (Figure 1B bottom row). When the cursor was within 1 cm of the target at the home position, the next target appeared. While participants were reaching the target at the home position, there was no time limit so that participants could start the next trial at their own pace. On the other hand, in the consecutive task, after each trial, the next target immediately appeared and started to move (Figure 1B top row). The consecutive task was adopted to examine task performance in a dynamic environment. Throughout the experiment, when the cursor was within 1 cm of the target, a pleasant chime sound was played from the earmuffs of the HMD (1,400 Hz tone, lasting for 200 ms), and the target color changed from pink to blue to signify success. Participants were instructed to achieve as much success as possible in each block. They were required to keep their feet flat on the force plate and their hands folded behind their back throughout the experiment. In addition, they were instructed to keep their feet positioned parallel and 30 cm apart, defined as the distance between the lateral edges of the feet. No specific instructions were provided regarding other movements.

### 2.4 Experimental procedure

The experiment consisted of three phases: a pre-single phase, a consecutive phase, and a post-single phase. The rationale for including both single and consecutive tasks was as follows. The single task ensured that every trial started from the same initial posture (the home position), which facilitated comparisons across trials and phases and allowed us to assess learning effects (see below). However, the same initial posture could not examine how varying initial conditions influence performance. The consecutive task addressed this limitation because participants were required to initiate each trial from various postures depending on the previous trial. This design enabled us to dissociate the effects of target position and initial body posture (details see Data analysis). Additionally, the order of presentation (single-consecutive-single) was fixed to avoid interference between task types. Specifically, randomizing the order of the task types could have confused participants due to their different task features (i.e., whether or not the cursor returned to the home position), so the task types were clearly separated into phases.

Before the pre-single phase, participants practiced controlling the cursor for 2–3 min, during which the participants could freely move the cursor. In the pre-single phase, participants completed a single task to familiarize themselves with the task and the weight of the HMD (600 g). A total of 400 targets were presented, and each trial's time constraint was 1.6 s. There were 6 blocks (75 trials in 1–5 blocks and 25 trials in 6 blocks). It takes 2–3 min to complete 75 trials. The duration of each block depended on the participant's pace, as there was no time limit while the target was in the home position in the single task.

In the consecutive phase, participants completed a consecutive task under three time constraint conditions (0.8, 1.2, and 1.6 s). 400 targets in each condition were presented. One block had different numbers of trials across time constraint conditions (1.6 s, 75 trials; 1.2 s, 100 trials; 0.8 s, 150 trials; Figure 1C). This ensured that each block lasted 2 min. The last blocks for 1.6 s condition and 0.8 s condition had 25 trials and 100 trials, respectively.

In the post-single phase, participants completed a single task again, allowing us to assess learning effects by comparing task performance between the pre-single phase and the post-single phase. We quantified learning effects to distinguish the effects of the postural demands of interest from repetition-related improvements.

Participants took rest breaks for 1 min between blocks and 3 min between phases. Additional rest time was allowed if the participants needed it. The experiment was interrupted if the participants' feet left the force plate, defined as a fall. If the experiment was interrupted, it was resumed after the feet were aligned with the marked positions. If a fall occurred while the target was moving, that trial was excluded from the analysis as an interrupted trial. However, trials were included if the fall occurred after the target had fully reached the cursor movement line. In addition, trials between the occurrence of a fall and the experimenter's interruption were also excluded as interrupted trials. As a result, 8 falls occurred, and a total of 22 trials were excluded in all participants and phases.

### 2.5 Data analysis

#### 2.5.1 Validity of experimental manipulation

We quantified postures across different target positions, and the cursor velocity and acceleration to confirm whether experimental manipulation modulated postural demands.

We confirmed whether target positions modulate postures. The whole body was approximated with three links (foot-pelvis, pelvis-thorax, thorax-head). The foot position was the mean position between both lateral malleoli (see Supplementary Materials). We then quantified the leaning angle of each link: the pelvis–foot angle was defined relative to vertical (upright), whereas the thorax–pelvis and head–thorax angles were defined relative to the distal link. We reported the leaning angle of each link when the target arrived at the cursor movement line.

We then confirmed whether time constraints modulate cursor accelerations. The cursor velocity and acceleration were calculated using a three-point differentiation algorithm after the cursor position data were low-pass filtered at 4 Hz using a zero-phase fourth-order Butterworth filter. Positive velocity and acceleration values indicated movement toward the target. The peak velocity and acceleration in each trial were quantified, and the mean peak value across trials was calculated for each participant and condition. Similarly, the peak velocity and acceleration of the mediolateral CoP data were calculated. We used CoP data as a supplementary measure, together with cursor movement and link angle data, to illustrate that participants exhibited substantial postural displacement rather than maintaining a near-upright posture throughout the task.

#### 2.5.2 Analysis of the effect of target positions

We investigated task performance in response to the postural demands. We quantified the error between the cursor and the target positions when the target arrived at the cursor movement line. For instance, positive values of errors indicated a smaller deviation from upright than that required by the target (i.e., undershooting). Negative values indicated the opposite (i.e., overshooting). Our study considered the errors as an index of task performance. Our interest is to quantify how target positions and time constraints (i.e., postural demands) affect errors. To isolate these effects, we also accounted for other factors that could affect errors, such as learning (see Experimental procedure) and movement distance (see below).

We first quantified errors across target positions in the single task and the 1.6-second condition of the consecutive task. In the single task, because the initial positions in reaching were identical across trials, there was a strong correlation between target positions and movement distances (defined by the distance between the target and the cursor when the target appeared). For example, when the target position is 10 cm, the movement distance is also 10 cm, because the initial position is always almost 0 cm. Thus, only the single task was not able to dissociate the effects of target positions and movement distances on errors. In contrast, in the consecutive task that had the same time constraints as the single task, target positions and movement distances are mutually independent. Even when the target position is 10 cm, the movement distance can be 3 cm if the initial position is 7 cm, depending on the previous trial. Hence, the consecutive task allowed us to examine the effect of target positions on errors, accounting for the effect of movement distances. First, to qualitatively investigate whether task performance can change in two task conditions, we compared the errors in response to target positions in the single task and the 1.6-second condition of the consecutive task. Then, to quantitatively examine the effect of target positions on errors while considering movement distances, we used statistical analysis as explained in the Statistical analysis section.

We then quantified a critical point at which the errors started to increase in response to target positions. The critical point was calculated for each participant as follows. First, we combined errors in response to the left and right target positions relative to the home position, as direction did not affect the errors. We then calculated the mean of errors for each target area. Finally, we defined the critical point as the target position for which the lower limit of the 95% confidence interval of the mean error remained greater than zero for all more lateral target positions.

Finally, as expected, some target areas were unreachable for every participant throughout the experiment (see Whole body task). To exclude the increase in errors due to each participant's spatial constraints (e.g., joint range of motion), all our analyses only included trials in which target positions were within each participant's cursor range of motion. The cursor range of motion was defined by the maximum lateral cursor displacement throughout the experiment. In addition, when reporting the group mean values and their 95% confidence intervals on errors or link angles, we restricted the analysis to the 15th target area. This was because data for the 16th and subsequent target areas were obtained from only four participants due to individual differences in the cursor range of motion. To mitigate the influence of individual variability on the mean calculation, we reported the mean error only for target areas up to the 15th, where the number of participants exceeded half of the total (*n* ≥ 12).

#### 2.5.3 Analysis of the effect of time constraints

To examine how the acceleration of deviation affects errors, we compared errors across different time constraint conditions. When comparing errors across conditions, we accounted for the effects of target positions and movement distances. Target positions were classified into five groups, each group consisting of three adjacent target areas. This classification was based on the fact that more than half of the participants (*n* ≥ 12) had a range of motion that included up to the 15th target area. Thus, we divided all target areas into five groups, each with three areas. The data after the 16th area was included in the 5th target position group. After classifying target positions, we then analyzed the effects of movement distance. To do so, we partitioned the distribution of movement distances into 5 bins for each condition and target group, ensuring that each bin contained approximately equal numbers of samples. Finally, we quantified the mean error for each bin to examine how movement distance influences errors. The above partition of target positions and movement distances was done for visualization purposes. Therefore, we performed statistical analysis on the effect of target positions and movement distances on errors across conditions as explained below.

Data analysis was performed offline using MATLAB (R2024b, The MathWorks Inc., Natick, MA, United States).

### 2.6 Statistical analysis

To test the idea that target positions and time constraints affect errors, we fitted a generalized linear mixed-effects model to the data from the consecutive task. The dependent variable was an error. Three independent variables were target position, time constraint, and movement distance. The model equation was:

$$\begin{array}{c} y∼\beta_{0}+\beta_{1}\mathrm{Target}+\beta_{2}\mathrm{Distance}+\beta_{3}(\mathrm{Target}\times \mathrm{Distance})+\beta_{4}\mathrm{Time}\\ +b_{0}+b_{1}\mathrm{Target}+b_{2}\mathrm{Distance}\\ y∼\mathrm{Normal} \end{array}$$

where *β* represents the fixed-effect coefficients and *b* represents the participant-specific random effects, including random intercepts and random slopes. To minimize the influence of physical or physiological factors, such as joint range of motion or insufficient strength to move quickly, which could contribute to error, the data used in the model were limited to trials where the target positions fell within each participant's range of motion of the cursor, and where the movement distance was less than the maximum distance covered by the cursor under each time constraint condition. Importantly, unlike the criteria used for data visualization in the figures, the statistical analysis included all trials within each participant's reachable range. Furthermore, to account for individual differences in physical or physiological capacity, the model included participant-specific random intercepts and slopes for target position and movement distance. A generalized linear mixed-effects model was conducted using lme4 package (20) in R (version 4.2.2) and RStudio (21, 22).

We conducted a multiple regression analysis using MATLAB to examine whether participants' height and mass accounted for individual differences in the critical point. We used the regression model with the critical point as the dependent variable and height and mass as independent variables.

In the statistical analysis, a *p*-value of less than 0.05 was considered statistically significant.

## 3 Results

Figure 2A illustrates a representative time series of the cursor and CoP movement for the 1.6-second time constraint condition during the consecutive phase. The CoP movement generally coincides with the cursor movement. Figure 2B shows the group means of the range of motion, peak velocity, and peak acceleration for the cursor movement for all conditions. As time constraints shortened, the range of motion was smaller (mean ± SE, single 1.6 s, 20.8 ± 0.60 cm; 1.6 s, 21.1 ± 0.68 cm; 1.2 s, 19.5 ± 0.56 cm; 0.8 s, 15.5 ± 0.59 cm). The peak velocity increased (single 1.6 s, 19.8 ± 0.75 cm/s; 1.6 s, 20.8 ± 0.80 cm/s; 1.2 s, 23.1 ± 0.90 cm/s; 0.8 s, 24.1 ± 1.48 cm/s). The peak acceleration increased (single 1.6 s, 58.3 ± 3.33 cm/s^2^; 1.6 s, 59.6 ± 2.68 cm/s^2^; 1.2 s, 73.7 ± 3.41 cm/s^2^; 0.8 s, 89.8 ± 6.00 cm/s^2^). Similarly, Figure 2C shows the group means of the range of motion, peak velocity, and peak acceleration for the CoP movement for all conditions. The range of motion was invariant for all conditions (mean ± SD, single 1.6 s, 12.6 ± 0.27 cm; 1.6 s, 12.8 ± 0.20 cm; 1.2 s, 12.6 ± 0.20 cm; 0.8 s, 12.4 ± 0.27 cm). The peak velocity increased as time constraints shortened (single 1.6 s, 39.6 ± 2.7 cm/s; 1.6 s, 39.2 ± 2.4 cm/s; 1.2 s, 47.8 ± 2.7 cm/s; 0.8 s, 58.7 ± 3.8 cm/s). The peak acceleration increased as time constraints shortened (single 1.6 s, 322.3 ± 24.8 cm/s^2^; 1.6 s, 313.4 ± 22.1 cm/s^2^; 1.2 s, 389.0 ± 25.7 cm/s^2^; 0.8 s, 544.6 ± 39.2 cm/s^2^).

**Figure 3:**
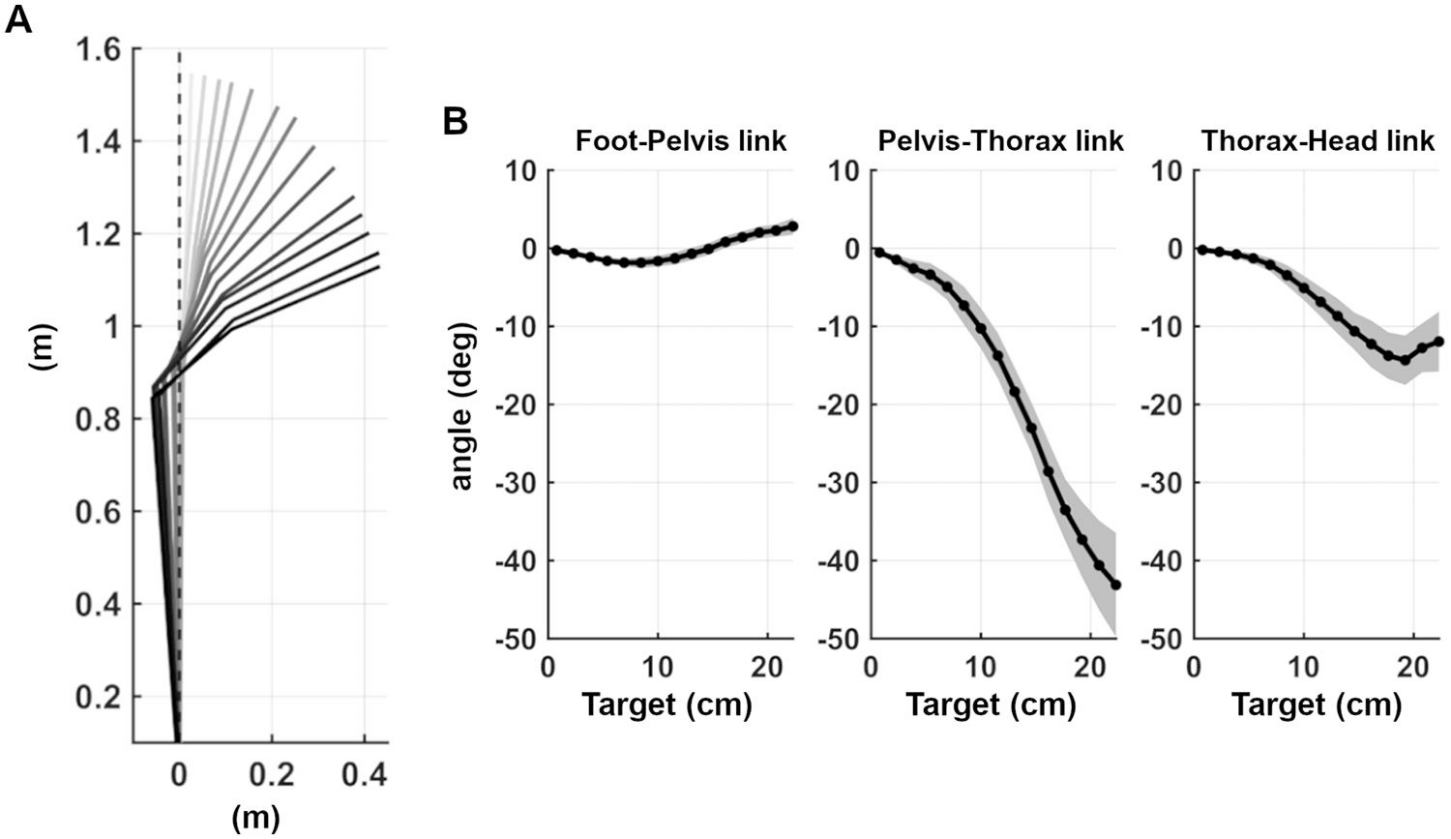
Postures across different target positions. **(A)** The difference in postural orientation across target positions in the single task during the post-single phase. Line darkness corresponds to different target positions. Three lines show three links: foot-pelvis, pelvis-thorax, thorax-head. **(B)** Group mean of the link angles in response to different target positions. Shaded areas represent the 95% confidence interval of the mean.

Figure 3A illustrates the difference in postures across target positions for a representative participant in the single task during the post-single phase. Figure 3B shows the group mean leaning angle of each link across target positions. The change in the foot-pelvis link angle was small across target positions. In contrast, the pelvis-thorax link and thorax-head link angles decreased, indicating that the links leaned more toward the target as the target positions became more lateral.

### 3.1 Effect of target positions on errors in a single task

**Figure 4:**
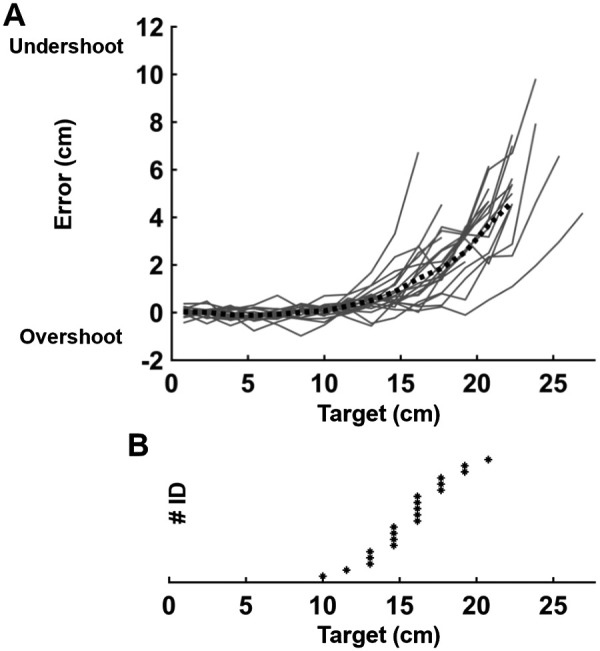
Task performance of the single task. **(A)** Error between the cursor and the target positions as a function of target position for each participant in the single task (post-single phase). Errors were calculated when the target reached the cursor movement line. Positive values indicate that the cursor was medial to the target (undershoot). Negative values indicate that the cursor was lateral to the target (overshoot). The dashed line represents the mean across all participants. Thin lines represent individual participants. **(B)** Asterisks denote the critical points at which the lower boundary of the 95% confidence interval exceeded zero for each participant.

To clarify how target positions affect errors between the cursor and target positions, we compared errors for different target positions within each participant's range of motion. Figure 4A shows errors in the single task during the post-single phase, where the time constraint was 1.6 s, and participants started reaching from an upright posture on all trials. We observed that errors increased as the target positions were lateral for all participants. Interestingly, despite being well before the cursor range of motion, there was a critical point after which the errors increased sharply. Furthermore, Figure 4B demonstrates that the critical points varied by participants, although the participants had similar physical properties (mean ± SD, height: 1.71 ± 0.01 m, body mass: 62.3 ± 5.9 kg). The multiple regression analysis revealed no significant effect of participants' height (*β* = -0.22 ± 0.46, *p* = 0.64) and mass (*β* = 0.026 ± 0.11, *p* = 0.82) on the critical point.

**Figure 5:**
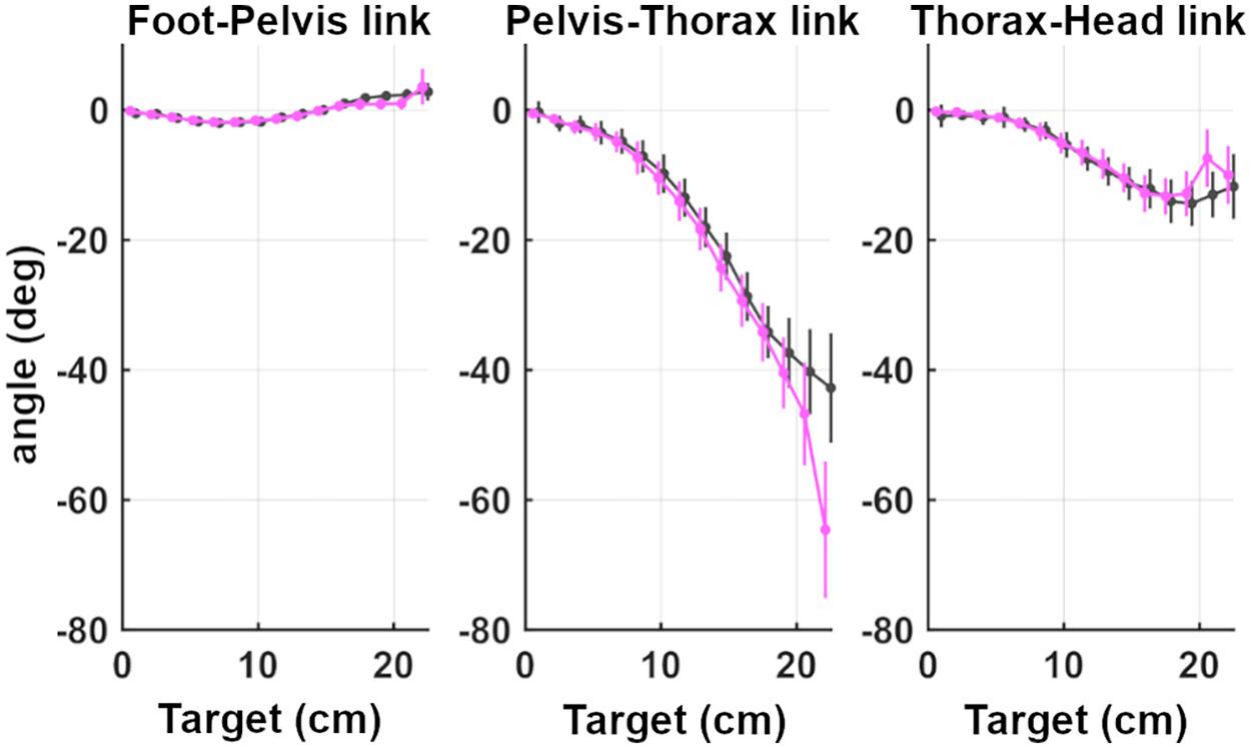
Postures across different target positions for successful and failed trials. Pink lines represent the group mean of link angles across target positions in successful trials, while black lines represent those in failed trials. Data are from the single-task condition during the post-single phase. Error bars indicate the 95% confidence interval of the mean. For visualization purposes, the *x*-axis positions of pink and black dots were slightly shifted.

To examine the differences in the whole-body coordination pattern when the error was below 1 cm (i.e., task success) or larger, we quantified the coordination relationships between three links (foot–pelvis, pelvis–thorax, thorax–head). As shown in Figure 5, when the target positions were small, there was no clear difference in the coordination pattern between successful trials (pink) and failed trials (black). In contrast, at more lateral target positions where errors increased, differences in the coordination pattern were observed, particularly in the pelvis-thorax link (middle panel). In addition, there was the trend that the participants adopted counter-rotational movements, in which the foot–pelvis link moved in the opposite direction to the other two upper-links, as target positions became lateral (Figure 3B). Figure 5 shows that the participants adopted counter-rotational movements regardless of task success or failure, indicating that counter-rotational movements are a robust strategy in the balance-challenging conditions rather than directly determining task success.

In addition, Supplementary Figure S1 shows the learning effect by comparing errors between the single tasks in the pre-single phase and the post-single phase. The more medial the target position, the more rapidly participants exhibited improvement in the first few trials of the pre-single phase. In contrast, the more lateral the target position, the slower the improvement was and continued throughout the experiment. Despite this slow learning effect, the increase in errors for lateral target positions observed in Figure 4A remained evident.

### 3.2 Effect of target positions on errors in consecutive tasks

**Figure 6:**
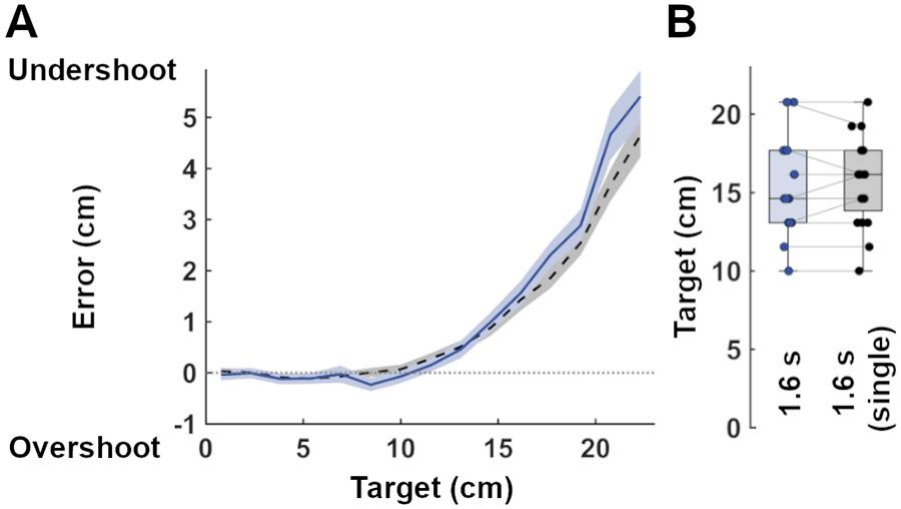
Comparison between the single task and the consecutive task. **(A)** Error as a function of target position. Lines and shaded areas represent the mean and 95% confidence interval across participants. The solid black line indicates the consecutive task (1.6-second time constraint condition), while the dashed line indicates the single task in the post-single phase. Data are shown only for target positions within the cursor range of motion for more than half of the participants (*n* ≥ 12). **(B)** Comparison of critical points. In the box plot, the midline, box size, and whiskers represent the median, 25th-75th percentiles, and the range within 1.5 times the interquartile range (IQR), respectively. Dots represent individual participants.

To examine how target positions affect errors for more dynamic postural control tasks, we compared errors between the 1.6-second condition of the consecutive task and the 1.6-second condition of the single task during the post-single phase. Figure 6A shows that the trend of errors was similar for two tasks. Moreover, Figure 6B demonstrates similar critical points between two tasks (mean ± SD, single 1.6 s, 15.6 ± 2.7 cm; consecutive 1.6 s, 15.7 ± 3.1 cm). In the single task, the initial postures were identical on all trials. Conversely, in the consecutive task, the initial postures varied depending on the previous trial. Thus, if target positions were the same, the initial postures differed between the two tasks, resulting in variations in movement distances from the initial cursor position to a target position. Despite these differences, we found the identical critical points between these tasks when the time constraint was 1.6 s, suggesting the effect of target positions on errors (Figure 6B).

### 3.3 Effect of time constraints on errors

**Figure 7:**
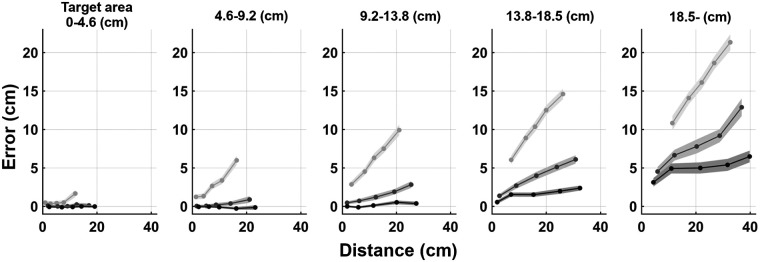
Relationship between movement distance and error for target areas and time constraint conditions. Error as a function of the movement distance between the target and cursor when the target appeared. Target positions were divided into 5 groups, each shown separately. Line darkness corresponds to different time constraint conditions. Shaded areas represent the 95% confidence interval of the mean. Data points are plotted at the center of distance bins, each adjusted to include approximately equal numbers of samples.

Next, we examined whether and how time constraints affected errors. Supplementary Figure S2 shows that, for all conditions, errors increased as target positions were lateral. However, as time constraints shortened, the critical point shifted toward the medial side (mean ± SD, 1.6 s, 15.7 ± 3.1 cm; 1.2 s, 12.1 ± 2.7, 0.8 s, 5.7 ± 1.9 cm), indicating that factors other than target positions affect errors. Thus, to examine the effect of time constraints on errors, we compared errors across different time constraint conditions by considering the effects of target positions and movement distances on errors. Figure 7 illustrates the relationship between errors and movement distances, presented separately for the five target area groups. First, as found in Figure 6, errors increased as target areas were lateral for all conditions. Second, errors increased as time constraints shortened for each target area group. Finally, for each target area group, errors increased as movement distance was greater, except for the most medial target area (Figure 5 most left). This result remained unchanged even when the minimal error within 0.5 s after the time constraint was re-quantified to account for timing errors.

To test the idea that target position, time constraint, and movement distance affect errors, we performed a generalized linear mixed-effects model. Significant main effects were observed in target position [*β* = 0.21, SE = 0.012, 95% CI = (0.19, 0.23), *p* < 0.001], time constraint [*β* = −0.049, SE = 0.00070, 95% CI = (−0.050, −0.047), *p* < 0.001], and movement distance [*β* = 0.066, SE = 0.0085, 95% CI = (0.049, 0.083), *p* < 0.001]. In addition, the interaction between target position and movement distance was significant [*β* = 0.0016, SE = 0.00048, 95% CI = (0.00067, 0.0026), *p* < 0.001].

Collectively, the three factors—target position, time constraint, and movement distance—influence errors in our task. It is important to note that target position and time constraint affect errors significantly, even when considering movement distance due to different initial postures in the consecutive task.

## 4 Discussion

The purpose of this study was to characterize task performance in response to varying postural demands, including maximal leaning postures. We manipulated postural demand by requiring various positions and accelerations of the upper body using different target positions and time constraints to reach a target. Throughout our experiment, there were eight falls among all participants and all conditions. In two of the eight falls, the participants took a step after successfully reaching the target. This indicates that our task required the participants to adopt maximal leaning postures, and in some cases, participants achieved the task goal even at the expense of maintaining standing. In addition, we observed that the upper body leaning angle increased as the target positions became more lateral (Figure 3), demonstrating increased destabilizing torque by gravity. The peak cursor accelerations increased as the time constraints shortened (Figure 2B), thereby associating with increased destabilizing torque. These results confirm that our experimental manipulations varied the postural demands through changes in target positions and time constraints. We quantified errors between the cursor and target positions as task performance. Our main finding was that target positions and time constraints significantly affect errors. Additionally, we observed a large individual difference in the critical point of target positions at which errors began to increase sharply. We will discuss below why errors increased as target positions became more lateral and time constraints shortened.

### 4.1 The increase in errors beyond the critical point

We observed significantly large errors beyond the critical point (Figures 4, 6). Moreover, the increased errors were associated with decreased leaning angles of the pelvis-thorax link (i.e., leaning angle of the upper body) (Figure 5). Importantly, the critical point was located within each participant's range of motion, indicating that the required posture itself remained mechanically achievable. Therefore, the abrupt increase in errors cannot be explained solely by the dynamics of a whole-body based on equations of motion, without considering biological factors such as neural control or muscle function. Below, we discuss possible factors underlying the increase in errors from the perspectives of central nervous system strategies and biomechanical limitations.

#### 4.1.1 The central nervous system's strategy

One possible mechanism for this behavior may be explained within the framework of optimal feedback control (23). We assume that the controlled plant is a multi-segment system, such as an inverted pendulum composed of two or three linked segments (24). Given this plant, gravity always acts on each segment, and thus the CNS must generate corrective torque to maintain standing. At the same time, when performing our experimental task, the CNS has to generate torque to achieve a goal-directed leaning. From this perspective, at least two possible interpretations may account for our results.

As the first case, we consider a conventional cost function combining endpoint error and effort (23–25), while satisfying the constraint that the center of the body's mass (CoM) remains within the base of support. First, the CoM constraint alone cannot account for the increased errors. Participants could reach targets beyond the critical point at least once, indicating that the required postures were mechanically feasible under the CoM constraint. Therefore, the cost function could be responsible for explaining the observed increased errors in this case. Endpoint error is defined as the cursor error measured experimentally. Effort is defined as the net torque required to lean and maintain a required posture that is consistent with the destabilizing torque. If the destabilizing torque in response to target positions has the same nonlinear trend as the trend of error in Figure 4, in other words, if there is a strong correlation between destabilizing torque and error, optimal feedback control could explain the observed increased errors due to a tradeoff between endpoint error and effort (23). This possibility may be testable in future studies by directly quantifying destabilizing torque through inverse dynamics and comparing a computational model with behavior. If increased errors are strongly correlated with destabilizing torque, it would provide empirical support for this explanation.

As another case, we consider a more flexible cost function including an additional term beyond endpoint error and effort. The additional term penalizes angular deviation of the CoM from the vertical axis only when that deviation exceeds a certain threshold. In this case, neither the CoM constraint by the BoS nor the correlation between destabilizing torque and error is necessary to explain the increased error. Unlike the first case, in which the destabilizing torque itself is related to the increased error, this case explains that the increased error could emerge due to the threshold in the cost function. Specifically, when the required angular deviation remains below a threshold, the CNS minimizes only goal-directed costs (e.g., endpoint error, effort), resulting in preserved endpoint accuracy before the critical point. However, once the required deviation exceeds the threshold, a fall-avoidance cost (i.e., penalizing the deviations from the vertical axis) is engaged, producing larger endpoint errors after the critical point. The idea of the fall-avoidance cost is consistent with earlier suggestion that the CNS considers the risk of injury, such as falls, in generating movements (2). Babič et al. (2) found that, in a motor adaptation task, the residual difference between the original and adapted trajectories was significantly larger in a whole-body task than in a seated arm-reaching task. They concluded that this difference was due to the CNS accounting for the risk of falling, such as the destabilizing effect of gravity. In addition to their suggestions, the idea of considering a fall-avoidance cost only when a certain threshold is exceeded may provide a possible interpretation consistent with both the previous finding and our observation. However, the existence of such a threshold-based cost function was not directly tested in the present study. Therefore, future experimental and computational studies will be necessary to clarify how the CNS balances goal-directed performance and fall avoidance.

Additionally, we observed that endpoint accuracy did not change across the target positions within a critical point (Figures 4, 6). In addition, no difference was found in whole-body coordination between successful and failed trials within a critical point (Figure 5). These results are also broadly consistent with previous works demonstrating a high adaptability of postural control against postural constraints. Several studies have manipulated postural constraints using approaches such as reducing the size of stability limits, requiring reaching beyond one's reach, and/or introducing an unstable support surface. These studies reported that endpoint accuracy (11–15, 26) and muscle coordination (27, 28) remained unaltered compared to less challenging conditions. This high adaptability against postural constraints may align with the consistent endpoint accuracy within the critical point observed in our study. Indeed, the constraints in previous studies were designed to prevent the CoP from approaching the stability limits (16), resulting in a narrower range of CoP movement than that observed in our task (Figure 2C). Thus, the leaning postures required in previous studies were likely below a critical point. Importantly, this behavior within a critical point can be explained by either formulation within the OFC framework described above.

Finally, coordination patterns across body segments are also important characteristics to reveal the CNS's control strategy. In our task, the participants modulated the coordination pattern from a strategy, in which all body segments leaned toward the targets, to counter-rotational movements, in which the lower body segment moved in the opposite direction to the upper body (Figure 3). Because the former strategy involves the CoM's significant displacement, this transition into the latter strategy may reflect an adaptive strategy to bring the upper-body close to the target and minimize the CoM displacement. Recently, it has been shown that the spectrum of coordination patterns is well explained by the difference in cost function (29). Therefore, in future research, it is important to develop models that can account for coordination patterns, in addition to task performance, in order to comprehensively understand postural control mechanisms.

#### 4.1.2 Biomechanical limitations

Another possible explanation for the increased errors beyond the critical point may be the biomechanical limitation, such as insufficient muscle strength. Previous studies have shown that the functional base of support decreases with age, due to reduced ankle strength and restricted joint range of motion (18). Another study highlighted the close relationship between muscle strength and the functional stability limit, particularly the role of toe flexor strength (19). Taken together, these findings suggest that muscle strength could constrain the maximum leaning angle. This biomechanical constraint provides a plausible explanation for the abrupt increase in errors beyond the critical point. However, the participants in our study were still able to maintain leaning postures (Figure 5, pink line) even beyond the critical point. This indicates that a lack of muscle strength alone cannot fully explain the variability observed across trials in our study.

In summary, our results demonstrate a threshold-like change in task performance: errors were preserved within the critical point but increased beyond it. This behavior may reflect either a CNS strategy, a biomechanical limitation, or their combined influence. To reveal the detailed mechanisms, future studies combining physiological measurements and computational modeling are required.

### 4.2 Individual differences in the critical point

We found a large individual difference in the critical points (Figures 4, 6) despite our participants having similar physical properties (height: 1.71 ± 0.01 m, body mass: 62.3 ± 5.9 kg, mean ± SD). Moreover, the multiple regression analysis revealed no significant effect of participants' height and mass on the critical point. These findings suggest that the individual difference in the critical points is shaped by the CNS control process rather than being determined only by physiological factors. Such individual differences in motor strategies have been explained in various ways. For instance, one study explained the differences in terms of variations in internal values (i.e., the relative weights in the cost function) (30). A higher penalty on the cost associated with task success (e.g., endpoint error) could lead to a critical point further away from the upright posture. Other studies have described individual differences in motor strategies as differences in risk sensitivity (31–35). Additionally, studies on postural threat have explained differences in postural strategies due to fear of falling (36, 37). If an individual estimates the risk of falling as high or perceives fear of falling highly, the critical point would likely be closer to the upright posture. Although it is challenging to identify the specific causes of the individual differences in our study, future research needs to examine these psychological and physiological factors, for example, by manipulating postural threat through whole-body goal-directed tasks at elevated heights.

### 4.3 The increase in errors in short time constraints

In our study, we also examined the effect of postural demands regarding accelerations by comparing errors across time constraint conditions. Our result shows that the errors increased significantly as time constraints shortened, even if the target positions and movement distances were identical across the conditions (Figure 7). This result indicates that the CNS did not generate the necessary movement velocity to reach the target. We suggest that the CNS might prefer to suppress the velocity or accelerations of deviation from upright at the expense of task success. Computational studies support this hypothesis, showing that quiet standing measured experimentally can be explained well by a postural control system using the CoM velocity information as a feedback variable (5, 6, 38). In the dynamic environment requiring high velocity and accelerations, more conservative postural control strategies may be adopted.

However, it is possible that an alternative explanation exists. Previous studies found that unidirectional CoP and CoM movements had a mean duration of around 1 s during quiet standing (39, 40). It is suggested that this reflects the characteristics of intermittent control by the CNS since the mean duration did not alter in response to different dynamics requirements (41, 42). On the contrary, in our study, the mean duration of CoP movements modulated in response to different time constraint conditions (0.8 s condition: 0.40 s, 1.2 s condition: 0.45 s, 1.6 s condition: 0.48 s, see Supplementary Figure S3). Therefore, the apparent limit of movement velocity may occur because the CNS considers the risk of falling based on the CoM velocity or acceleration.

### 4.4 Limitations and implications for broader populations

The present study included only young, healthy male participants, which limits the generalizability of our findings. Older adults and patients with neurological disorders such as Parkinson's disease often report greater fear of falling (43, 44), which may shift the critical point closer to the upright posture compared to healthy young adults. Investigating these populations in future studies would clarify the mechanism of falling prevention.

### 4.5 Implications for rehabilitation and real-world applications

The present findings suggest that postural control strategies may not be uniform across all body states but instead depend on the level of postural demand. This finding could underscore the need to examine whether rehabilitation and training conducted in upright postures can generalize to more posturally challenging conditions. Incorporating such conditions into rehabilitation programs, sports training, and the design of assistive technologies (e.g., exoskeletons and robotic devices) may therefore be critical for improving safety and performance in daily life.

## 5 Conclusion

In conclusion, this study examined task performance in response to various postural demands. The results demonstrated that target positions, time constraints, and the movement distances influenced the task performance, quantified as the positional error between the cursor and the target. The error increased sharply when the required target positions exceeded an individual-specific threshold. Moreover, the error increased as the time constraints shortened and the movement distances increased, especially when the target positions were located more laterally relative to the upright posture. These findings demonstrate that reaching accuracy was maintained under low to modest postural demands but deteriorated once postural demands became high. Such insights could have important implications for understanding the control mechanisms during goal-directed whole-body movements.

## Data Availability

The original contributions presented in the study are included in the article/Supplementary Material, further inquiries can be directed to the corresponding author.
