# Supplementary material for: Reaching accuracy declines with postural demand during whole-body leaning
Source: Front Sports Act Living. 2026 Jun 9;8:1843450. doi: 10.3389/fspor.2026.1843450 (PMC13286970; doi:10.3389/fspor.2026.1843450)
Supplement: Supplementary file 1 [file Presentation1.pdf]

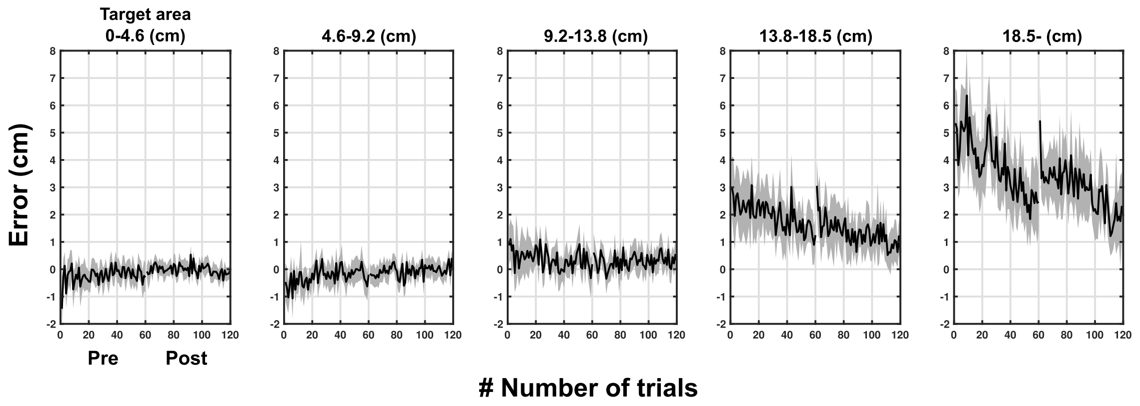

S1 Fig. Learning effect.

To examine the learning effect, we compared errors between the single tasks in the pre-single and post-single phases. Target positions were classified into five groups, as in Fig 5, and we obtained the time course of errors for each target area group and each participant. Furthermore, we calculated the mean time course of errors across participants. Left and right target positions were combined relative to the home position. Consequently, each target area group included 120 trials across the pre-single and post-single phases. Time courses of errors were displayed separately. The black line represents the mean error across participants. Shaded area represents the 95% confidence interval. Each phase (pre-single and post-single) included 60 trials.
