# Supplementary material for: Reaching accuracy declines with postural demand during whole-body leaning
Source: Front Sports Act Living. 2026 Jun 9;8:1843450. doi: 10.3389/fspor.2026.1843450 (PMC13286970; doi:10.3389/fspor.2026.1843450)
Supplement: Supplementary file 2 [file Presentation2.pdf]

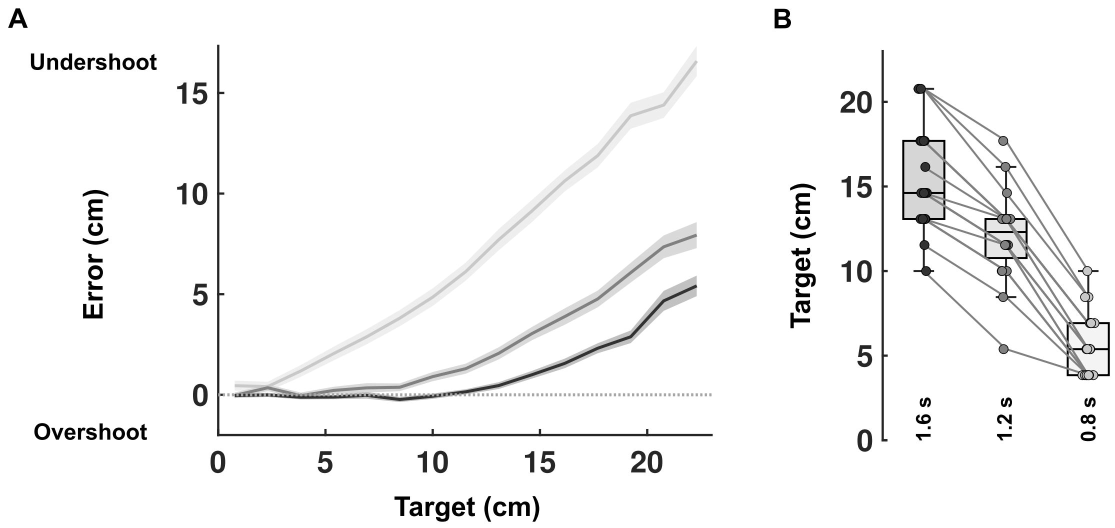

S2 Fig. Task performance for time constraint conditions.

(A) Error as a function of target position. Lines and shaded areas indicate the mean and 95% confidence interval across participants. Line darkness corresponds to different time constraint conditions. Data are shown only for target positions within the cursor range of motion for more than half of the participants ($n \geq 12$). (B) Comparison of the critical points. In the box plot, the midline, box size, and whiskers indicate the median, 25th-75th percentiles, and the range within 1.5 times the interquartile range (IQR), respectively. Points represent individual participants.
