# Supplementary material for: Reaching accuracy declines with postural demand during whole-body leaning
Source: Front Sports Act Living. 2026 Jun 9;8:1843450. doi: 10.3389/fspor.2026.1843450 (PMC13286970; doi:10.3389/fspor.2026.1843450)
Supplement: Supplementary file 3 [file Presentation3.pdf]

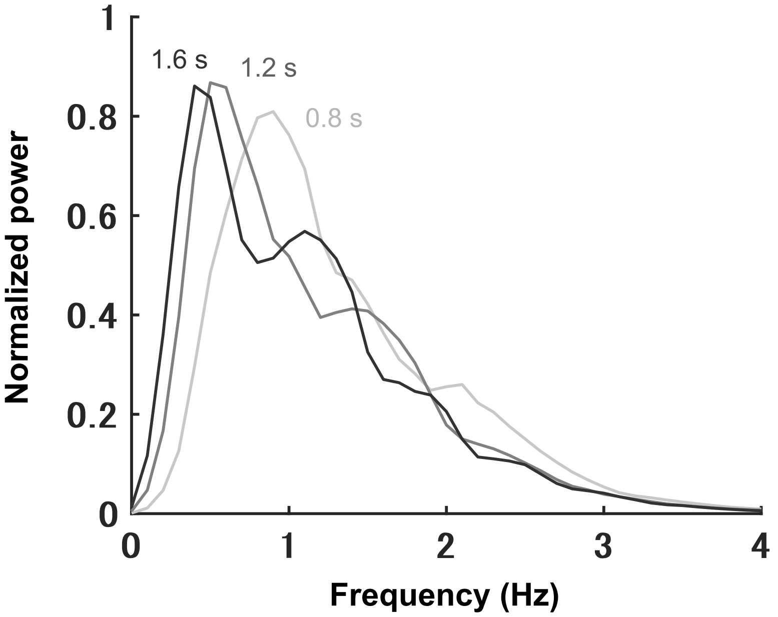

S3 Fig. Mean duration for time constraint conditions.

Based on previous studies [1, 2], we calculated the mean duration of CoP. We calculated power spectra for CoP velocity via Welch's method using the MATLAB function *pwelch* with 10000-sample long windows with an overlap of 50% and a Hamming window. Thus, we obtained a resolution of 0.1 Hz, consistent with previous studies [1, 2].

For each block in each time constraint condition, all power spectra were divided by the peak power in the range 0–3 Hz. We averaged all blocks per participant within each condition, and then averaged across participants. We calculated the mean frequency within 0–3 Hz bandwidth for each block using the following equation:

$$\overline{f} = \frac{\sum f P_{vv}}{\sum P_{vv}}$$

where f is the frequency and P_{vv} is the velocity power spectrum. This value represents the mean frequency of the CoP velocity. Each velocity cycle contains two unidirectional oscillations. Thus, the mean duration, $\overline{T}$, is calculated using the following equation:

$$\overline{T} = \frac{1}{2\overline{f}}$$

where $\overline{f}$ is the mean frequency of the CoP velocity. The power spectrum of CoP velocity was averaged across all participants. Power spectra were normalized to their maximal values. Line darkness corresponds to different time constraint conditions.

Reference

1. Loram ID, Maganaris CN, Lakie M. Human postural sway results from frequent, ballistic bias impulses by soleus and gastrocnemius. J Physiol. 2005;564(Pt 1):295-311. Epub 20050120. doi: 10.1113/jphysiol.2004.076307. PubMed PMID: 15661824.

2. Loram ID, Gawthrop PJ, Lakie M. The frequency of human, manual adjustments in balancing an inverted pendulum is constrained by intrinsic physiological factors. J Physiol. 2006;577(Pt 1):417-32. Epub 20060914. doi: 10.1113/jphysiol.2006.118786. PubMed PMID: 16973712.
