# Supplementary material for: Reaching accuracy declines with postural demand during whole-body leaning
Source: Front Sports Act Living. 2026 Jun 9;8:1843450. doi: 10.3389/fspor.2026.1843450 (PMC13286970; doi:10.3389/fspor.2026.1843450)
Supplement: Supplementary file 4 [file Presentation4.pdf]

Supplementary Materials

CoP data calculation

The CoP data was calculated based on the three components of the ground reaction force (F_x, F_y, and F_z) and moment (M_x, M_y, and M_z) that were stored on a computer via a 16-bit A/D converter (NI-DAQ USB-6229, National Instruments, Austin, TX). The CoP data were low-pass filtered at 10 Hz.

VR setup

The VR environment was created using Unity (version 2019.3.9f1; Unity Technologies, California).

Motion capture setups

In addition to the six body landmarks, rigid bodies were also attached to both lateral malleoli. Each rigid body was created by four or five infrared reflective markers. The marker positions were sampled at 100 Hz using a 3D optical motion capture system (OptiTrack V100, Natural Point Inc., Oregon, USA). The motion data were streamed in real-time from Motive 2.0.2 software (Natural Point Inc., Oregon, USA) via NatNet SDK to LabVIEW (National Instruments, Austin, TX). The motion data were low-pass filtered at 4 Hz.
